# Predictors of the Onset of Manic Symptoms and a (Hypo)Manic Episode in Patients with Major Depressive Disorder

**DOI:** 10.1371/journal.pone.0106871

**Published:** 2014-09-26

**Authors:** Lynn Boschloo, Annet T. Spijker, Erik Hoencamp, Ralph Kupka, Willem A. Nolen, Robert A. Schoevers, Brenda W. J. H. Penninx

**Affiliations:** 1 University of Groningen, University Medical Center Groningen (UMCG), Interdisciplinary Center Psychopathology and Emotion Regulation (ICPE)/University Center Psychiatry (UCP), Groningen, The Netherlands; 2 Vrije University Medical Center (VUMC), Department of Psychiatry and Extramuraal Geneeskundig Onderzoek (EMGO) Institute for Health and Care Research, Amsterdam, The Netherlands; 3 PsyQ, Department of Mood Disorders, The Hague, The Netherlands; 4 Parnassia Group, The Hague, The Netherlands; 5 Leiden University, Institute of Psychology, Leiden, The Netherlands; 6 Leiden University Medical Center, Department of Psychiatry, Leiden, The Netherlands; University of Iowa Hospitals & Clinics, United States of America

## Abstract

**Objective:**

One third of patients with a major depressive episode also experience manic symptoms or, even, a (hypo)manic episode. Retrospective studies on the temporal sequencing of symptomatology suggest that the majority of these patients report depressive symptoms before the onset of manic symptoms. However, prospective studies are scarce and this study will, therefore, prospectively examine the onset of either manic symptoms or a (hypo)manic episode in patients with a major depressive disorder. In addition, we will consider the impact of a large set of potential risk factors on both outcomes.

**Methodology:**

Four-year follow-up data were used to determine the onset of manic symptoms as well as a CIDI-based (hypo)manic episode in a large sample (n = 889, age: 18–65 years) of outpatients with a major depressive disorder and without manic symptoms at baseline. Baseline vulnerability (i.e., sociodemographics, family history of depression, childhood trauma, life-events) and clinical (i.e., isolated manic symptoms, depression characteristics, and psychiatric comorbidity) factors were considered as potential risk factors.

**Results:**

In our sample of depressed patients, 15.9% developed manic symptoms and an additional 4.7% developed a (hypo)manic episode during four years. Baseline isolated manic symptoms and comorbid alcohol dependence predicted both the onset of manic symptoms and a (hypo)manic episode. Low education only predicted the onset of manic symptoms, whereas male gender, childhood trauma and severity of depressive symptoms showed strong associations with, especially, the onset of (hypo)manic episodes.

**Conclusions:**

A substantial proportion (20.6%) of patients with a major depressive disorder later developed manic symptoms or a (hypo)manic episode. Interestingly, some identified risk factors differed for the two outcomes, which may indicate that pathways leading to the onset of manic symptoms or a (hypo)manic episode might be different. Our findings indirectly support a clinical staging model.

## Introduction

Converging evidence from community and clinical studies support the high prevalence of manic symptoms as well as (hypo)manic episodes in patients with a major depressive episode [1]–[2]. For example, Angst et al. [2] showed that 35% of depressed patients reported manic symptoms and an additional 12% met criteria for a (hypo)manic episode. The clinical relevance of bipolarity has motivated the DSM-5 work group to not only include bipolar disorders but also a mixed features specifier for major depressive disorder in the new version of the DSM [3]. As bipolar disorder often starts with depressive episodes [4], this implies that the initial presence of a major depressive episode in the absence of manic symptoms or a (hypo)manic episode may be a critical phase and could therefore be a potential target for early intervention strategies.

As much is still unclear about the underlying processes, an essential next step would be to identify risk factors for the onset of manic symptoms as well as a (hypo)manic episode in depressed patients. Sociodemographics (e.g., male gender [5]–[6]) and other vulnerability factors (e.g., childhood trauma [7]) have shown to be more strongly related to bipolar disorder than to major depressive disorder. In addition, manic symptoms [1], [8], depression characteristics (e.g., severity [10]–[11]) and psychiatric comorbidity (e.g., anxiety disorders or alcohol dependence [6]) are clinical factors that have been associated with bipolarity in depressed patients. As only few prospective studies have considered both the onset of manic symptoms and the onset of a (hypo)manic, it is still unclear whether risk factors differ for these outcomes.

Therefore, the present study focuses on the onset of manic symptoms as well as a (hypo)manic episode during four years of follow-up in a large sample of patients with a lifetime major depressive disorder and so far without significant lifetime manic symptoms at baseline (n = 889). We will examine whether several vulnerability and clinical factors *independently* predict the onset of manic symptoms or a (hypo)manic episode and it was, additionally, tested whether risk factors differ for these two outcomes.

## Methods

### Study sample

Data were derived from the Netherlands Study of Depression and Anxiety (NESDA), an ongoing cohort study aimed at examining the long-term course and consequences of depressive and anxiety disorders in the adult (18–65 years) population. A total of 2,981 persons were included at the baseline assessment in 2004–2007, consisting of healthy controls (22%) and patients with a lifetime depressive and/or anxiety disorder (78%). To represent various settings and stages of psychopathology, persons were recruited from the community (19%), primary care (54%) and outpatient mental health care services (27%). Community-based participants had previously been identified in a population-based study; primary care participants were identified through a three-stage screening procedure (involving the Kessler-10 [11] and the short-form Composite International Diagnostic Interview [CIDI] psychiatric interview by phone) conducted among a random sample of patients of 65 General Practitioners; and mental health care participants were recruited consecutively when newly enrolled at one of the 17 participating mental health organization locations. Persons with insufficient command of the Dutch language or a primary clinical diagnosis of bipolar disorder, obsessive compulsive disorder, severe substance use disorder, psychotic disorder or organic psychiatric disorder, as reported by them or their (mental) health practitioner, were excluded. A detailed description of the NESDA study design and sampling procedures can be found elsewhere [12]. The research protocol was approved by the Ethical Committee of participating universities and all participants provided written informed consent.

The baseline assessment consisted of an extended face-to-face interview, including a standardized diagnostic psychiatric interview, and paper-and-pencil questionnaires. For the present study, we selected patients with a lifetime DSM-IV major depressive disorder (n = 1,925), as assessed with the CIDI (version 2.1) at baseline. The CIDI is highly reliable and valid [13] and was administered by specially trained research staff. To examine the onset of manic symptoms as well as a (hypo)manic episode, we aimed to exclude all patients with significant lifetime manic symptoms at baseline. Unfortunately, the CIDI section on bipolar disorders was not conducted at baseline and, consequently, it was not possible to determine whether patients met criteria for a DSM-IV bipolar disorder at baseline. However, patients with a self-reported or health care professional-reported primary clinical diagnosis of bipolar disorder had already been excluded for the initial participation in NESDA. In addition, we excluded all patients with lifetime manic symptoms (n = 581) as assessed with the Mood Disorder Questionnaire (MDQ [14]–[15]) or with missing data on this measure (n = 274) at baseline. The MDQ is a fifteen-item self-report questionnaire comprising thirteen dichotomous items on the lifetime presence or absence of manic symptoms as well as two additional questions regarding the clustering of symptoms in time and severity of related problems. We considered lifetime manic symptoms to be present when a patient reported at least seven positive answers from the thirteen items, irrespective of the answers on the two additional questions. Our group [16] recently showed that this cut-off is especially adequate in detecting a recent (i.e. past two years) (hypo)manic episode (sensitivity  = 0.83 and specificity  = 0.82).

In addition to the baseline assessment, follow-up assessments were conducted after two years (overall response rate: 87.1% [17]) and four years (overall response rate: 80.6%). In the subsample of 1,070 participants with a lifetime MDD and without lifetime manic symptoms at baseline, 181 (16.9%) did not provide complete data at the two-year or four-year follow-up assessments, resulting in a sample of 889 patients for the current analyses. Non-responders were significantly less educated (11.7 versus 12.4 years of education, p = 0.01) compared to responders, whereas gender (p = 0.47) and age (p = 0.45) were not associated with non-response.

### Measures

#### Outcome variables

In our sample, we considered the onset of manic symptoms as well as a (hypo)manic episode during four years of follow-up. The onset of manic symptoms was based on the presence of seven or more positive answers on the MDQ at the two-year or four-year follow-up assessment, in the absence of a (hypo)manic episode. The onset of a (hypo)manic episode was based on the presence of a DSM-IV hypomanic or manic episode as assessed with the CIDI section on bipolar disorders at the two- or four-year follow-up assessment.

#### Baseline predictors

On the basis of previously reported associations with manic symptoms or a (hypo)manic episode, an extensive set of potential predictors (i.e., vulnerability and clinical factors) was selected.

1. Vulnerability factors included sociodemographics, family history of depressive disorder, childhood trauma and life-events at baseline.
  - Sociodemographics included gender, age (in years) and education (in years).
  - Family history of a unipolar or bipolar depressive episode was assessed with the family tree method [18], which obtains information on the presence of psychiatric disorders among first-degree relatives (not including offspring of the patient).
  - Based on the Childhood Trauma Inventory, a cumulative childhood trauma index (range: 0–8) was constructed considering the frequency (never  = 0, once/sometimes  = 1, often or more  = 2) of emotional neglect, psychological abuse, physical abuse and sexual abuse before the age of 16 years [19].
  - A total count of twelve negative life events in the past year was constructed using Brugha's List of Threatening Experiences [20].
2. Clinical factors included isolated manic symptoms, various depression characteristics and psychiatric comorbidity at baseline.
  - The number of isolated manic symptoms was defined as the count of the thirteen dichotomous MDQ items on the lifetime presence or absence of manic symptoms. As patients with a total score of seven or higher were already excluded (see sample description), the number of isolated manic symptoms ranged from zero to six.
  - From the depression characteristics, we first considered the age (in years) of onset of major depressive disorder as based on CIDI questions regarding the onset of a lifetime diagnosis at the baseline assessment. We also considered whether patients had experienced recurrent episodes versus a single episode during their life. The severity of depressive symptoms in the week prior to baseline was defined as the total score on the 30-item self-report Inventory of Depressive Symptomatology (IDS) [21]. We also determined whether the disorder had atypical features as assessed by specific IDS items. The atypical subtype was defined as having mood reactivity and at least three of the following: increased appetite, hypersomnia, leaden paralysis, and interpersonal rejection sensitivity [22]. Sleep duration was based on the average number of hours of sleep per night during the month prior to baseline by distinguishing short (≤6 hours) versus normal (>6 hours) duration.
  - Psychiatric comorbidity was assessed by the CIDI interview separately considering lifetime diagnoses of any anxiety disorder (i.e., generalized anxiety disorder, social phobia, panic disorder, and agoraphobia) and alcohol dependence.

### Statistical analyses

Analyses were conducted using SPSS version 20 statistical software (SPSS Inc, Chicago, Illinois). In our sample of patients with major depressive disorder and without significant lifetime manic symptoms, we first determined the onset of manic symptoms and a (hypo)manic episode. Then, descriptive statistics were used to summarize the sample characteristics across these groups. To identify independent risk factors (i.e., vulnerability and clinical factors) of each outcome, multinomial logistic regression analyses were performed in which predictors with a p≤0.05 in univariable analyses were entered into one multivariable analysis. To provide insight into the nature of these associations, results of the univariable as well as multivariable analyses were shown. Additional logistic regression analyses were performed to test whether predictors showed differential associations with the onset of manic symptoms versus a (hypo)manic episode. To be able to compare odds ratios across all continuous factors, odds ratios with 95% confidence intervals were expressed per standard deviation increase.

## Results

### Sample characteristics

**Figure 1:**
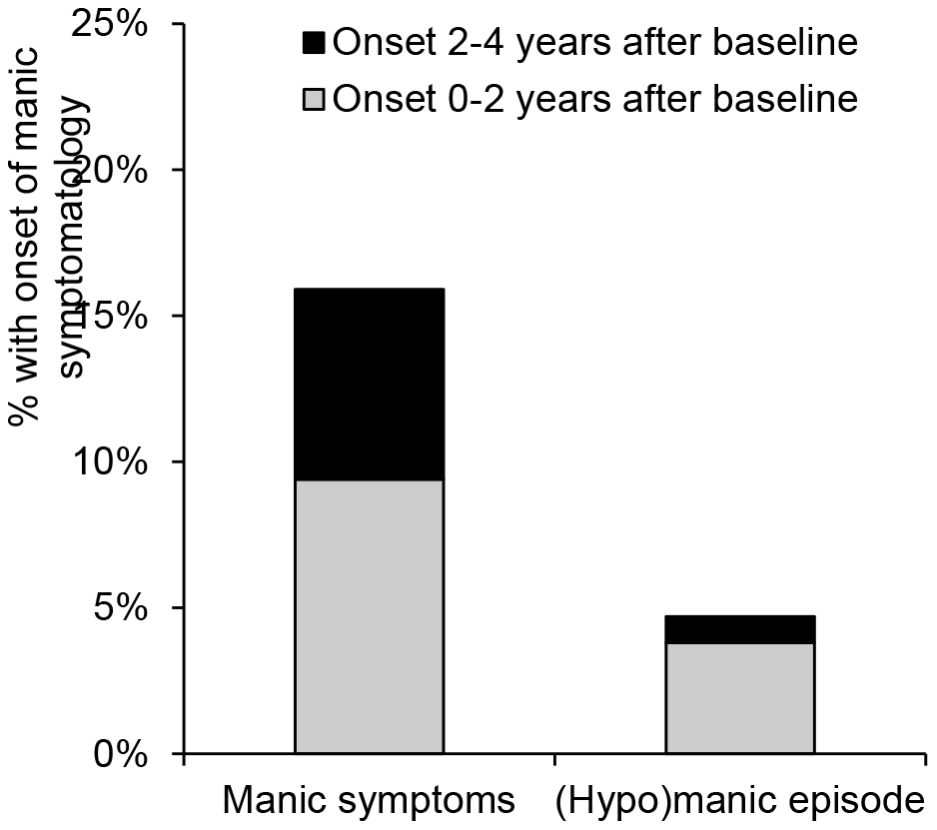
The onset of manic symptoms as well as a (hypo)manic episode during four years of follow-up.

In our sample of patients with lifetime major depressive disorder and without significant lifetime manic symptoms at baseline (n = 889), the mean age was 43.0 (SD = 12.4; range  = 18–65) years and 26.9% were men. Figure 1 shows that the onset was 15.9% (n = 141) for manic symptoms (i.e., at least seven positive items on the MDQ) and 4.7% (n = 42) for a DSM-IV hypomanic (n = 17) or manic (n = 25) episode during four years of follow-up. To provide more information about prodromal stages of bipolar disorder patients with a (hypo)manic episode during follow-up were asked about their age during which they first developed several manic symptoms. The onset of manic symptoms preceded the onset of a (hypo)manic episode with an average of 12.7 (SD = 13.9) years. This also implies that the majority (69%) of patients with a (hypo)manic episode reported the onset of first manic symptoms before the baseline assessment. Among patients with only manic symptoms but no (hypo)manic episodes during follow-up, no information about the age of onset of their first manic symptoms was available as this was not considered in the MDQ.

### Predictors of the onset of manic symptoms or a (hypo)manic episode

Descriptive statistics (Table 1) are presented in order to explore the role of vulnerability and clinical factors as predictors of the onset of manic symptoms and a (hypo)manic episode, respectively. To identify independent risk factors, a multivariable multinomial logistic regression analysis was performed including all predictors with a p≤0.05 in the univariable analyses (both results are presented in Table 2).

**Table 1:** Sample characteristics.

		Onset of manic symptomatology	
		Manic	(Hypo)manic
	Unipolar depression	symptoms	episode
	(n = 706)	(n = 141)	(n = 42)
	Mean (sd)/%	Mean (sd)/%	Mean (sd)/%
**Vulnerability factors**			
Gender (male)	23.8%	35.5%	57.1%
Age in years	43.1 (12.6)	42.3 (11.9)	43.5 (11.2)
Education in years	12.6 (3.2)	11.4 (3.1)	11.8 (3.5)
Family history of depressive disorder (yes)	71.7%	78.7%	78.6%
Severity of childhood trauma	1.6 (2.0)	1.8 (2.2)	2.9 (2.3)
Number of life events	0.8 (1.0)	0.8 (1.0)	0.9 (1.2)
**Clinical factors**			
Number of manic symptoms	3.0 (1.9)	4.3 (1.7)	4.2 (1.6)
Age of onset of MDD	28.5 (12.6)	28.2 (12.2)	28.2 (12.3)
Recurrent MDD (yes)	53.4%	50.4%	45.2%
Severity of depressive symptoms	21.7 (12.9)	26.2 (13.0)	33.4 (11.7)
Atypical depression subtype (yes)	9.6%	12.1%	26.2%
Short sleep duration (yes)	30.9%	30.5%	50.0%
Anxiety disorder (yes)	65.0%	69.5%	71.4%
Alcohol dependence (yes)	11.8%	19.9%	26.2%

**Table 2:** Vulnerability and clinical factors predicting the onset of manic symptoms as well as a (hypo)manic episode during four-year follow-up.

	Unadjusted a						Adjusted b						
	Manic symptoms			(Hypo)manic episode			Manic symptoms			(Hypo)manic episode			
	OR	95%CI	p	OR	95%CI	p	OR	95%CI	p	OR	95%CI	p	p^c^
**Vulnerability factors**													
Gender (male)	1.76	1.20–2.59	0.004	4.27	2.26–8.06	<0.001	1.54	1.01–2.34	0.04	5.17	2.52–10.59	<0.001	0.01
Age in years^d^	0.94	0.79–1.13	0.50	1.03	0.76–1.41	0.83	-	-	-	-	-	-	-
Education in years^d^	0.68	0.56–0.82	<0.001	0.79	0.57–1.08	0.13	0.77	0.62–0.94	0.01	1.00	0.72–1.41	0.98	0.13
FH of depressive disorder (yes)	1.46	0.95–2.26	0.09	1.45	0.68–3.08	0.34	-	-	-	-	-	-	-
Severity of childhood trauma^d^	1.09	0.91–1.31	0.33	1.74	1.33–2.28	<0.001	0.99	0.81–1.20	0.88	1.53	1.13–2.08	0.005	0.03
No. of life events^d^	1.02	0.86–1.23	0.80	1.10	0.82–1.48	0.52	-	-	-	-	-	-	
**Clinical factors**													
No. of isolated manic symptoms^d^	2.13	1.72–2.63	<0.001	2.06	1.44–2.95	<0.001	1.98	1.60–2.48	<0.001	1.74	1.17–2.60	0.006	0.90
Age of onset of MDD	0.97	0.81–1.17	0.76	0.97	0.71–1.33	0.86	-	-	-	-	-	-	
Recurrent MDD (yes)	0.89	0.62–1.27	0.51	0.72	0.39–1.35	0.31	-	-	-	-	-	-	
Severity of depressive symptoms^d^	1.42	1.18–1.69	<0.001	2.34	1.72–3.18	<0.001	1.33	1.06–1.68	0.01	1.85	1.24–2.74	0.002	0.048
Atypical subtype (yes)	1.29	0.73–2.26	0.38	3.33	1.60–6.92	0.001	0.73	0.38–1.42	0.35	1.60	0.63–4.04	0.32	0.34
Short sleep duration (yes)	0.98	0.66–1.45	0.93	2.24	1.20–4.19	0.01	0.70	0.45–1.08	0.11	1.15	0.57–2.33	0.70	0.28
Anxiety disorder (yes)	1.23	0.83–1.81	0.31	1.35	0.68–2.67	0.40	-	-	-	-	-	-	-
Alcohol dependence (yes)	1.86	1.16–2.98	0.01	2.66	1.29–5.50	0.008	1.74	1.05–2.90	0.03	1.80	0.81–4.01	0.15	0.94

a =  Unadjusted analyses, based on univariable multinomial logistic regression analyses.

b =  Adjusted analyses, based on multivariable multinomial logistic regression analyses including all variables that had p≤0.05 in the univariable analyses.

c =  Based on multivariable logistic regression analyses directly comparing patients developing manic symptoms with patients developing a (hypo)manic episode, including all variables that had p≤0.05 in the univariable analyses.

d =  For continuous variables, ORs are presented per SD increase.

#### Vulnerability factors

From the sociodemographics, male gender was a significant risk factor for the onset of both manic symptoms and a (hypo)manic episode, even after adjustment for other important vulnerability and clinical factors. Logistic regression analyses additionally showed that this association was stronger for a (hypo)manic episode than for manic symptoms. Low education was only independently related with manic symptoms, whereas childhood trauma was only independently associated with a (hypo)manic episode. None of the other vulnerability factors predicted the onset of manic symptomatology.

#### Clinical factors

**Figure 2:**
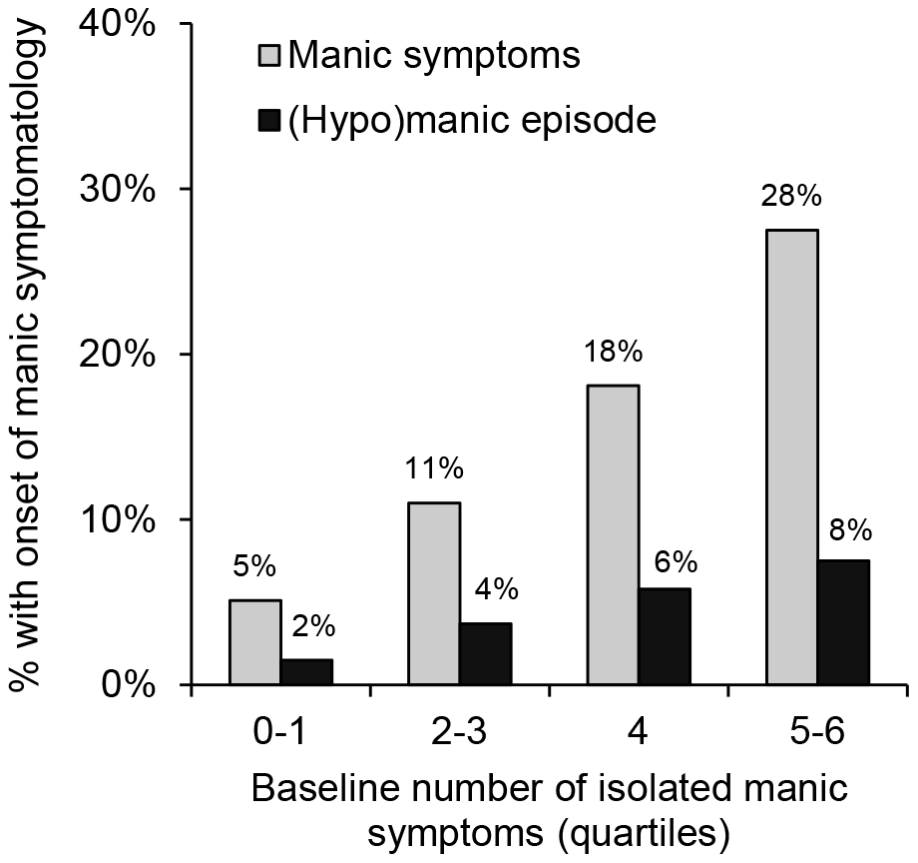
Baseline number of isolated manic symptoms predicting the onset of manic symptoms as well as a (hypo)manic episode during four years of follow-up.

**Figure 3:**
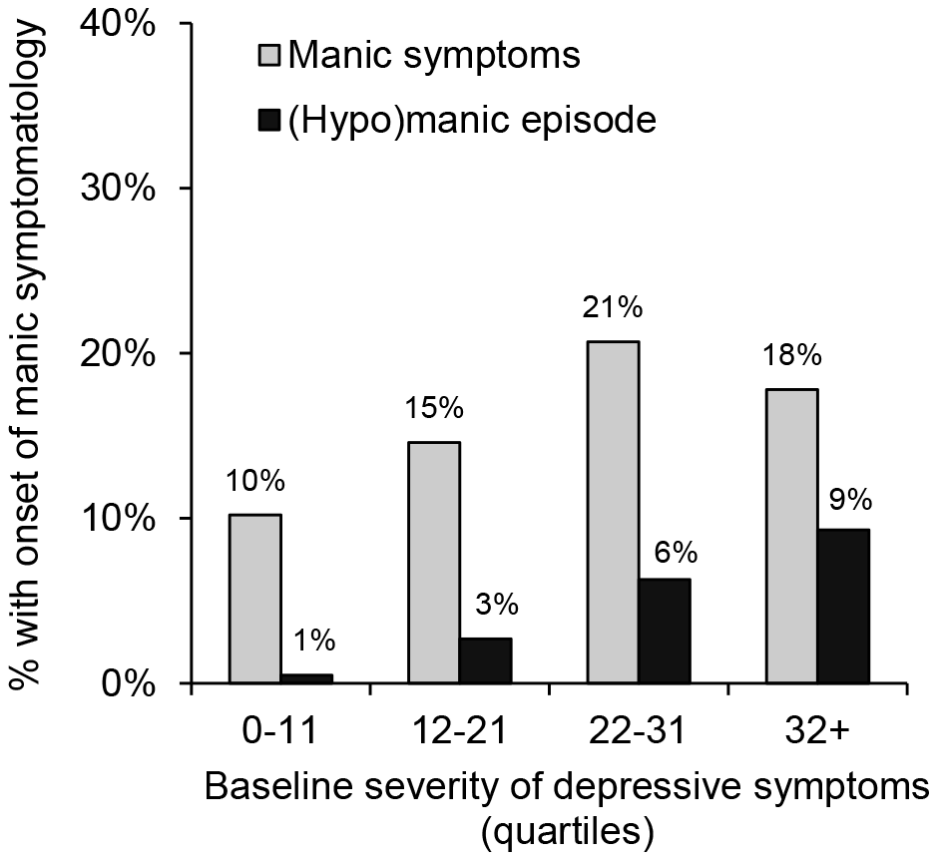
Baseline severity of depressive symptoms predicting the onset of manic symptomatology during four years of follow-up.

The total number of isolated manic symptoms (i.e., no more than six positive items on the MDQ) was a strong and independent predictor of the onset of manic symptoms as well as a (hypo)manic episode. These associations were similar for both outcomes, which was also illustrated in Figure 2 showing comparable dose-response relationships. In addition, severity of depressive symptoms was an independent predictor of both manic symptoms and (hypo)manic episodes. However, logistic regression analyses additionally showed that this association was stronger for the onset of a (hypo)manic episode compared to manic symptoms (see also Figure 3). Atypical features of depression and a short sleep duration were related to the onset of a (hypo)manic episode in the univariable but not the multivariable analyses, whereas none of the other depression characteristics showed a significant association with the onset of manic symptomatology. Comorbid alcohol dependence, but not a comorbid anxiety disorder, was a significant predictor of both outcomes in the univariable analyses. Although this association remained significant for the onset of manic symptoms in the multivariable analyses, the association with the onset of a (hypo)manic episode only reached a borderline significance level.

## Discussion

### Main findings

The present study aimed to improve our knowledge about the onset of manic symptoms as well as (hypo)manic episodes in patients with a lifetime major depressive disorder and without significant lifetime manic symptoms at baseline. During the subsequent four years, 15.9% of patients had developed manic symptoms and an additional 4.7% had developed a DSM-IV hypomanic or manic episode. Our study also identified several independent risk factors for the onset of manic symptoms and a (hypo)manic episode and showed that some of these factors played differential roles for the two outcomes.

### Clinical staging models for bipolar disorders

Our study showed that a substantial proportion (i.e., 20.6%) of patients with a diagnosis of major depressive disorder developed manic symptoms or a (hypo)manic episode during the subsequent four years. The presence of isolated manic symptoms was the most important risk factor for both outcomes, which is in line with a recent study demonstrating that these symptoms are a precursor of a later (hypo)manic episode [8]. In addition, patients who had developed a (hypo)manic episode during follow-up retrospectively reported that the presence of manic symptoms had preceded the onset of the full-blown episode with an average of thirteen years. These findings support a clinical staging model in which manic symptoms may increase over time and eventually result in a (hypo)manic episode [23]–[26].

Our findings further showed that depression severity was an important risk factor for the onset of manic symptoms and, especially, a (hypo)manic episode. This is in line with previous studies on bipolar disorders [2], [9]–[10] and, again, supports a clinical staging model in which patients with a major depressive disorder, and especially those who have progressed to a more severe subtype, have an increased risk of developing manic symptoms or a (hypo)manic episode [23]–[26]. This was also indirectly supported by our finding that comorbid alcohol dependence was a risk factor for both outcomes. The presence of alcohol dependence is known to be especially high in severe subtypes of major depressive disorder [27] and, as expected, reported associations with manic symptoms or a (hypo)manic episode decreased substantially after adjustment for baseline depression severity. Nonetheless, comorbid alcohol dependence could be an important indicator for both outcomes and screening instruments, such as the Alcohol Use Disorders Identification Test [28]–[29], may help to identify these high risk patients.

### Onset rates of (hypo)manic episodes

In our sample of depressed outpatients, an average of 1.2% had developed a (hypo)manic episode per year during follow-up. This annual onset rate is smaller than, for example, the 2.8% that was observed in depressed outpatients in Finland [10] and the 3.1% in hospitalized inpatients in the United States [30]. These contrasting findings may be explained by methodological differences. Our study, for example, excluded all patients with significant manic symptoms at baseline, as assessed with a screening instrument for manic symptoms. In contrast, the two previous studies excluded patients with a (hypo)manic episode, as assessed with a clinical interview, but did not consider baseline manic symptoms [10], [30]. Consequently, these samples may have included some patients with subthreshold manic symptoms, which could have resulted in higher onset rates of (hypo)manic episodes.

In addition, primary diagnoses of obsessive compulsive disorder, severe substance use disorder or psychotic disorder were all exclusion criteria for our study, but not for the two previous studies. As some of these factors might be risk factors for the onset of manic symptoms or a (hypo)manic episode [8], [30], onset rates in these studies are likely to be higher. An additional methodological difference is that our sample consisted of adults with a mean age of 43.0 years, which is comparable to the 40.2 years in the study of Holma et al. [10] but is much older than the 23.0 years in the sample of Goldberg et al. [30]. Although the majority of bipolar disorders typically develops earlier in life (i.e., before the age of 30 years), an additional peak of onset rates has been observed between 35 and 45 years of age [31]. The development of a (hypo)manic episode in our study should therefore be considered as a late-onset (hypo)manic episode and may be different from (hypo)manic episodes that develop earlier in life [31]–[32] (see also the ‘Risk factors’ section).

### Risk factors

Our study is unique in simultaneously examining the independent effects of several clinical as well as vulnerability factors on the onset of manic symptoms versus a (hypo)manic episode. From the clinical factors, the number of isolated manic symptoms and comorbid alcohol dependence tend to show similar associations with the onset of manic symptoms and a (hypo)manic episode, which may indicate that these factors play similar roles. However, the severity of depressive symptoms may be more important for the onset of a (hypo)manic episode compared to manic symptoms as the association with the first was stronger than with the latter. A similar pattern was found for male gender, as a vulnerability factor, which was an independent risk factor for the onset of manic symptoms and, especially, for a (hypo)manic episode. This is in line with previous studies showing that patients with increasing manic symptomatology were more often male [1], [5]–[6], [33]. We also found that childhood trauma independently predicted the onset of a (hypo)manic episode, which corroborates previous cross-sectional findings [7], but not the onset of manic symptoms. In contrast, low education predicted the onset of manic symptoms, but not a (hypo)manic episode. Although more research is needed, these findings are intriguing as they may indicate that processes underlying the onset of manic symptoms or the onset of a (hypo)manic episode might be different.

Our study also showed that family history of depressive disorders as well as an early onset of MDD were not related to the onset of manic symptoms or a (hypo)manic episode. This contradicts the findings of previous studies showing strong associations between these factors and bipolar disorder [8]–[10]. As genetic vulnerability may be more important for early-onset compared to late-onset bipolarity [31]–[32], this may explain the non-significant findings of our study mainly focussing on late-onset manic symptoms or a (hypo)manic episode. Future studies including both younger and older patients are necessary to determine whether etiological pathways for the development of early- versus late-onset manic symptoms or a (hypo)manic episode indeed differ.

### Strengths and limitations

Our study has both strengths and limitations. Methodological strengths are that we were one of the first studies that could prospectively examine the onset of manic symptoms as well as a (hypo)manic episode. In addition, we included a large sample (n = 889) of patients with a DSM-IV diagnosis of major depressive disorder without lifetime manic symptoms at baseline and tested the independent effects of several potential risk factors. Although we assessed various potential predictors of manic symptomatology, we did not have information about some well-known risk factors such as a family history of bipolar disorder [8]–[9] or psychotic symptoms [8], [30]. Although these factors may be less important for late-onset manic symptoms or (hypo)manic episodes (i.e. focus of our study) [31]–[32], we would like to encourage future researchers to gather this information, also in older samples of depressed patients.

Another important limitation is that the CIDI section on bipolar disorders was not conducted at baseline and, consequently, information about DSM-IV (hypo)manic episodes was not available at that time. However, patients with a baseline self-reported or health care professional-reported diagnosis of bipolar disorder were excluded from the study as well as patients with significant lifetime manic symptoms as assessed with the MDQ. Previous studies have shown mixed results regarding the validity of the MDQ (see [34] for a thorough review of the literature) and these inconsistencies might have resulted from recall bias (a patient's recall of recent symptoms is likely to be more reliable than recall of symptoms earlier in life). This was also demonstrated by a recent study of our group, in which we showed that the MDQ was adequate for detecting recent (hypo)manic episodes but not for lifetime episodes [16]. Consequently, our sample may have included some patients with a history of manic symptoms or, even, a (hypo)manic episode and this might have impacted our results. On the other hand, our sample may be similar to depressed patients in clinical practice as a history of manic symptoms or a (hypo)manic episode is often not recognized by patients or health care professionals [35]–[36]. The time lag between initial help seeking and the correct diagnosis is often more than ten years [37], [38] and, therefore, misdiagnosis of bipolar disorder is a major problem for both clinicians and researchers.

## Conclusions

In conclusion, 15.9% of patients with a major depressive disorder had developed manic symptoms and an additional 4.7% had developed a (hypo)manic episode during four years of follow-up. The number of isolated manic symptoms at baseline was an important, independent predictor of both stages in the development of bipolar disorder. This study also identified several other independent risk factors and demonstrated that some risk factors were different for the two outcomes.
